# Protective Action of Cannabidiol on Tiamulin Toxicity in Humans—In Vitro Study

**DOI:** 10.3390/ijms252413542

**Published:** 2024-12-18

**Authors:** Eryka Pankowska, Oliwia Kończak, Paula Żakowicz, Tatiana Wojciechowicz, Maciej Gogulski, Lidia Radko

**Affiliations:** 1 Students Scientific Society of Veterinary Medicine, Section of Veterinary Pharmacology and Toxicology “Paracelsus”, Faculty of Veterinary Medicine and Animal Sciences, Poznan University of Life Sciences, 60-637 Poznan, Poland; eryka.pankowska1@gmail.com (E.P.); 70907@student.puls.edu.pl (O.K.); paulazakowicz@gmail.com (P.Ż.); 2 Department of Animal Physiology, Biochemistry and Biostructure, Faculty of Veterinary Medicine and Animal Sciences, Poznan University of Life Sciences, 60-637 Poznan, Poland; tatiana.wojciechowicz@up.poznan.pl; 3 Department of Preclinical Sciences and Infectious Diseases, Faculty of Veterinary Medicine and Animal Sciences, Poznan University of Life Sciences, 60-637 Poznan, Poland; maciej.gogulski@up.poznan.pl

**Keywords:** veterinary drug, cannabidiol, interaction, human

## Abstract

The growing awareness and need to protect public health, including food safety, require a thorough study of the mechanism of action of veterinary drugs in consumers to reduce their negative impact on humans. Inappropriate use of veterinary drugs in animal husbandry, such as tiamulin, leads to the appearance of residues in edible animal tissues. The use of natural substances of plant origin, extracted from hemp (*Cannabis sativa* L.), such as cannabidiol (CBD), is one of the solutions to minimize the negative effects of tiamulin. This study aimed to determine the effect of CBD on the cytotoxicity of tiamulin in humans. The cytotoxic activity of tiamulin and the effect of its mixtures with CBD were tested after 72 h exposure to three human cell lines: SH-SY5Y, HepG2 and HEK-293. Cytotoxic concentrations (IC_50_) of the tested drug and in combination with CBD were assessed using five biochemical endpoints: mitochondrial and lysosomal activity, proliferation, cell membrane integrity and effects on DNA synthesis. Oxidative stress, cell death and cellular morphology were also assessed. The nature of the interaction between the veterinary drug and CBD was assessed using the combination index. The long-term effect of tiamulin inhibited lysosomal (SH-SY5SY) and mitochondrial (HepG2) activity and DNA synthesis (HEK-293). IC_50_ values for tiamulin ranged from 2.1 to >200 µg/mL (SH-SY5SY), 13.9 to 39.5 µg/mL (HepG2) and 8.5 to 76.9 µg/mL (HEK-293). IC_50_ values for the drug/CBD mixtures were higher. Reduced levels of oxidative stress, apoptosis and changes in cell morphology were demonstrated after exposure to the mixtures. Interactions between the veterinary drug and CBD showed a concentration-dependent nature of tiamulin in cell culture, ranging from antagonistic (low concentrations) to synergistic effects at high drug concentrations. The increased risk to human health associated with the presence of the veterinary drug in food products and the protective nature of CBD use underline the importance of these studies in food toxicology and require further investigation.

## 1. Introduction

Food safety is one of the most important issues raised by various scientific or social circles. Efforts are being made to find solutions that ensure food is free from contamination or minimize its negative impact on consumer health. A significant concern is the interactions between xenobiotics present in food (e.g., veterinary drugs, pesticides, mycotoxins, heavy metals, etc.) and dietary supplements, which are often used by individuals for prophylactic or therapeutic purposes.

**Figure 1:**
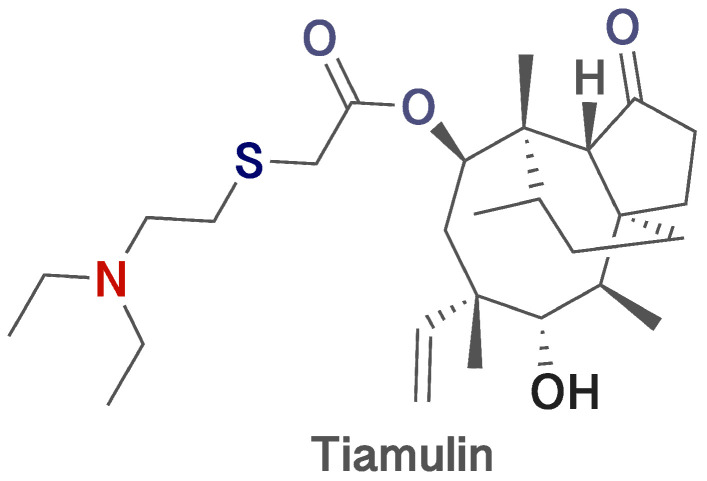
Structure of tiamulin (made using ChemDraw Pro 8.0 free software).

Pleuromutilins, natural antibiotics with a combined structure of 5-6-8 tricyclic diterpenoids (Figure 1) were first isolated from *Clitophilus scyphus* in 1951 [1]. They are used both in veterinary medicine (tiamulin and valnemulin) and in human medicine (retapamulin and lefamulin). Tiamulin is widely used in the treatment of lung and gastrointestinal diseases mainly in pigs and poultry (food-producing animals) caused by various bacterial agents [2,3,4]. The drug is most often administered in drinking water, is rapidly absorbed and then metabolized in the liver and excreted in urine [2]. In case of overdose or long-term use of tiamulin, liver disorders (increased activity of liver enzymes), kidney disorders (decreased urine production) and neurologic dysfunction by sedatives in animals have been observed [5]. A significant problem in animal husbandry is that due to the widespread use of tiamulin, high levels of it are detected in animal products, which may pose a risk to public health due to residues of this antibiotic. Consumers of animal products containing tiamulin residues may experience side effects [4]. Long-term consumption of tiamulin, especially in large quantities, can cause gastrointestinal problems [6], allergic reactions [7], increase the toxicity of other drugs [8] and contribute to the development of antibiotic resistance in humans [9,10].

**Figure 2:**
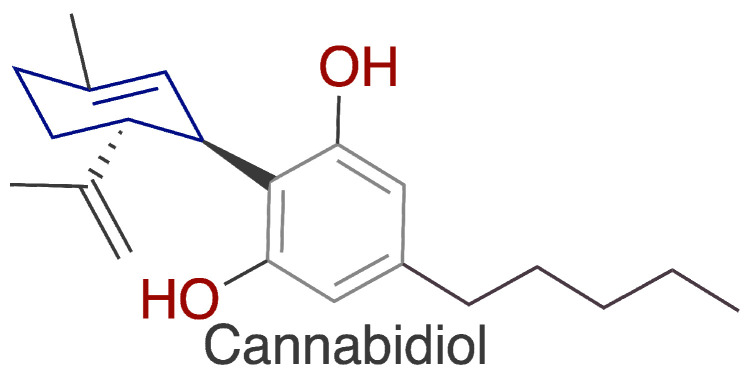
Structure of cannabidiol (made using ChemDraw Pro 8.0 free software).

To minimize this risk, many countries have set allowable residue limits for tiamulin in animal products (e.g., meat, milk, eggs) and have established withdrawal periods (7 days) to prevent excessive consumer exposure [11,12,13]. Despite these legal protections, the risk of exposure still exists. Therefore, efforts are being made to reduce the occurrence of side effects by exploring the use of biologically active plant-based substances. In recent years, hemp-based products have become very popular, as evidenced by the high sales of such supplements. Cannabidiol (CBD) (Figure 2) is one of the natural components of hemp (*Cannabis* L). Unlike THC, another compound in this group, CBD does not have psychoactive effects and is not addictive, which enhances its safety for use. Currently, numerous studies are being conducted worldwide to explore the medical applications of cannabinoids [14]. Due to its documented anti-inflammatory, antioxidant and protective properties, CBD is being investigated as a potential remedy to mitigate the harmful effects of toxic substances and drugs [15,16,17]. Research has shown that cannabidiol has broad protective effects in cases of toxic damage to the liver [18,19,20], kidneys [21,22,23], and nervous system [24,25,26].

Three human cell lines were used in this study as models of neuronal cells (SH-SY5Y line), liver cells (HepG2 line) and kidney cells (HEK-293 line). The SH-SY5Y cell line is a widely used human-derived neuroblastoma cell line. These cells are of neuronal origin and are often used in scientific research to study various aspects of neuroscience, including neurodegenerative diseases, cell signaling and drug development [27]. These cells are employed in screening potential neurotoxic compounds or evaluating drug candidates for central nervous system-related disorders [28]. The HepG2 cell line is one of the most commonly used cell models in cytotoxicity studies, particularly in the context of testing chemicals, drugs and other compounds that may affect the liver. HepG2 cells are derived from human liver cancer (hepatoma) and exhibit many of the functional characteristics of liver cells, making them a valuable tool in toxicity and metabolism studies [29,30]. Although SH-SY5Y or HepG2 cells are useful in many studies, they have certain limitations. For example, their cancerous origin means that they do not fully replicate all aspects of normal neuronal function or the complex in vivo environment. However, in in vitro studies, they are valuable cell models that allow for the determination of concentration ranges or mechanism of substance action.

The research objective of this innovative scientific project was to verify the effect of the active substance derived from hemp (*Cannabis sativa* L.)—cannabidiol (CBD)—at two non-toxic concentrations, 1.56 µg/mL (T + C1) and 3.125 µg/mL (T + C2), on the cytotoxic effect of a veterinary drug from the pleuromutilin group (tiamulin), the presence of which is found in food of animal origin, posing a risk to consumers. Thanks to this study on three cell lines of human origin: nerve cells (SH-SY5Y), liver cells (HepG2) and kidney cells (HEK-293), a lot of in-depth data were obtained to explain the effect of cannabidiol on the neuro-, hepato- and nephrotoxicity of the veterinary drugs tested. For this purpose, a series of tests were conducted to assess the cytotoxicity of the veterinary drugs and its mixtures with CBD in terms of inhibition of mitochondrial and lysosomal activity, synthesis of DNA, proliferation and cell membrane integrity on the cell cultures used. Additionally, oxidative stress, cell death and cell morphology were assessed. Based on the results obtained for the tested cellular parameters, the interactions occurring in the tested cell cultures were assessed. To the best of our knowledge, the conducted studies are innovative because the protective effect of cannabidiol against the cytotoxicity of tiamulin has not yet been investigated.

## 2. Results

### 2.1. The Action of Tiamulin and Its Mixtures with CBD on Human Cells

**Figure 3:**
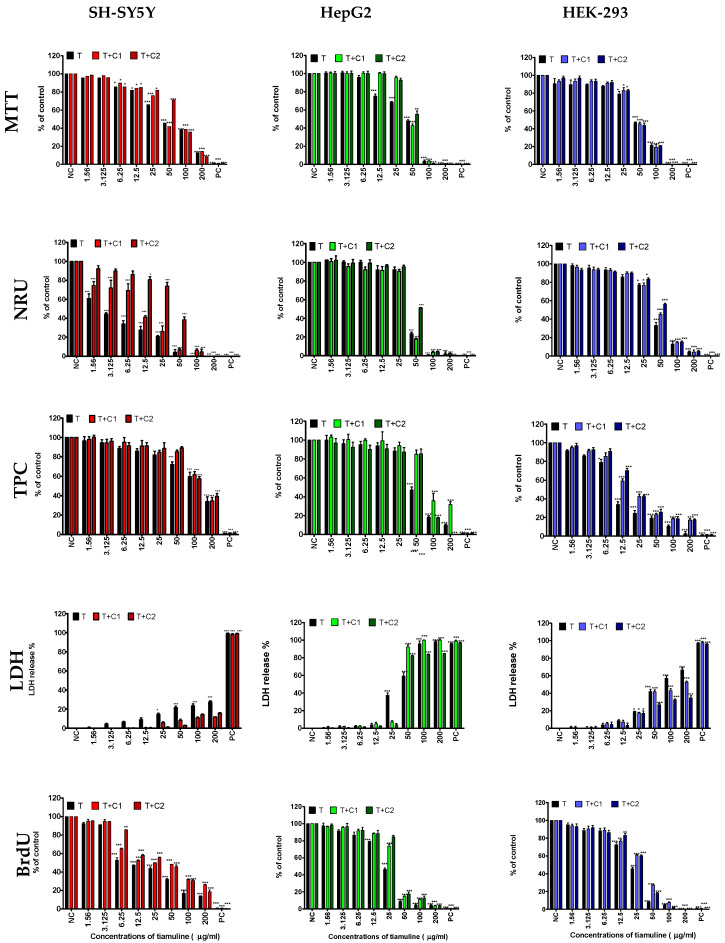
The effects of tiamulin and its co-action with cannabidiol at concentrations of 1.56 µg/mL (T + C1) and 3.12 µg/mL (T + C2) for the human SH-SY5H, HepG2, HEK-293 cell lines was evaluated using five endpoints: mitochondrial activity (MTT assay), lysosomal activity (NRU assay), proliferation (TPC assay), cell membrane integrity (LDH assay) and DNA synthesis (BrdU assay); data are presented as mean ± SEM (n = 3); NC (negative control); PC (positive control); (* *p* ≤ 0.05, ** *p* < 0.01, *** *p* < 0.001).

The cytotoxic potential of the veterinary drug and its mixtures with cannabidiol at two non-toxic concentrations: 1.56 and 3.15 µg/mL, on the human SH-SY5Y, HepG2 and HEK 293 cell lines was assessed by decreased viability of the cells within five biochemical endpoints: mitochondrial activity and lysosomal activity (MTT assays, reducing the tetrazolium dye MTT 3-(4,5-dimethylthiazol-2-yl)-2,5-diphenyltetrazolium bromide and neutral red uptake (NRU) assays), total protein content (TPC assay), cellular membrane integrity (leakage lactate dehydrogenase (LDH) assay) and DNA synthesis (the bromodeoxyuridine/5-bromo-2′-deoxyuridine incorporation into the DNA cells (BrdU) assay) after 72 h exposition. Data obtained from the measurement of the optical density in these assays were transformed to percentages in relation to the negative control group (0.1% DMSO in medium), considered to be 100% for the MTT, NRU, TPC and BrdU assays and 0% for the LDH assay. 1% Trition-X 100 solution in medium was used as the positive controls, (Figure 3).

#### 2.1.1. Cytotoxicity of Tiamulin in the Studied Cell Cultures

Tiamulin significantly (*p* ≤ 0.05) inhibited the lysosomal activity of SH-SY5Y cells from the low concentration of 1.56 µg/mL. At the concentration of 6.25 µg/mL, it inhibited mitochondrial activity and DNA synthesis. At higher concentrations, 25 µg/mL, disintegration of the cellular membrane cells and decreased total protein content in cell culture were observed (Figure 3).

At higher concentrations, tiamulin reduced the viability of HepG2 cells compared to SH-SY5Y nerve cells. The mechanism of action of the drug differed between the two cell types. In the case of HepG2 cells, a low concentration of 12.5 µg/mL of tiamulin inhibited mitochondrial activity. An increase in the drug concentration to 25 µg/mL resulted in the inhibition of DNA synthesis and disintegration of the cell membrane (Figure 3).

At a concentration of 50 µg/mL, tiamulin inhibited both lysosomal activity and cell proliferation (Figure 3). In a similar concentration range to HepG2 cells, tiamulin reduced the viability of HEK-293 cells. However, the mechanism of action of the drug differed between the two cell types. Tiamulin at a concentration of 12.5 µg/mL inhibited DNA synthesis and the proliferation of HEK-293 cells. Higher concentrations of the drug (25 µg/mL) inhibited mitochondrial and lysosomal activity and caused disintegration of the cell membrane (Figure 3).

#### 2.1.2. The Effects of CBD on the Cytotoxicity of the Tiamulin

Exposure of SH-SY5Y cells to the drug mixture containing CBD caused an inhibitory effect on cell proliferation and disintegration of the cell membrane at high concentrations of both mixtures—100 µg/mL and above 200 µg/mL, respectively. In contrast, inhibition of lysosomal activity occurred after exposing the cells to the T + C2 mixture with tiamulin at a concentration of 12.5 µg/mL (Figure 3). In the case of both mixtures of CBD with tiamulin at a concentration of 50 µg/mL, this caused the inhibition of mitochondrial activity and disintegration of the HepG2 cell membranes. On the other hand, mixtures containing tiamulin at a concentration of 100 µg/mL inhibited the proliferation of HepG2 cells (Figure 3). No effect on HEK-293 cell viability was observed with either of the two CBD–tiamulin mixtures with regards to the tested endpoints, compared to the veterinary drug alone (Figure 3).

#### 2.1.3. The IC50 Values for the Drug and Its Mixtures with CBD Obtained in the Studied Cell Cultures

Analysis of the IC_50_ values for tiamulin and its two mixtures with CBD (T + C1 and T + C2) on SH-SY5Y nerve cells showed that the IC_50_ values were lowest in the NRU and BrdU assays compared to the MTT, TPC and LDH assays. However, in both tests, a proportional increase in the IC_50_ values of the drug was observed as the CBD concentration in the mixture increased. In the MTT and TPC assays, a significant increase in the IC_50_ values was observed in the T + C2 mixture compared to the IC_50_ of the drug alone and the T + C1 mixture. In the LDH assay, the concentration values (IC_50_) of the drug and its two mixtures with CBD exceeded the highest tested concentration (<200 μg/mL) (Table 1).

Analysis of the IC_50_ values for tiamulin and its two mixtures with CBD (T + C1 and T + C2) on HepG2 liver cells revealed that the lowest IC_50_ values were observed in the BrdU assay, compared to the MTT, NRU, TPC and LDH assays. In the BrdU and TPC assays, a statistically significant increase in the IC_50_ values was observed for both mixtures. In the MTT and NRU assays, a significant increase in the IC_50_ values was observed only for the T + C2 mixture. In the LDH assay, no significant differences were found between the IC_50_ values for the drug and either of its mixtures (T + C1 and T + C2) (Table 1).

Analysis of the IC_50_ values for tiamulin and its two mixtures with CBD (T + C1 and T + C2) on HEK-293 kidney cells revealed that the lowest IC_50_ values were observed in the TPC assay, compared to the MTT, NRU, LDH and BrdU assays. In the TPC and BrdU assays, a statistically significant increase in the IC_50_ values was observed for both mixtures. In contrast, in the NRU and LDH assays, the IC_50_ values of the drug increased in proportion to the concentration of CBD in the mixture. In the MTT assay, no significant differences were observed between the IC_50_ values for tiamulin and its two mixtures (T + C1 and T + C2) (Table 1).

### 2.2. Interaction of Tiamulin with Cannabidiol

The types of interactions between tiamulin and cannabidiol, in relation to cytotoxicity against SH-SY5Y, HepG2 and HEK-293 cells, were analyzed by calculating combination index (CI) values. Synergism was defined as CI < 0.9, an additive effect as 0.9 < CI < 1.1 and antagonism as CI > 1.1. Two mixtures of the veterinary drug with cannabidiol were tested: one with a low CBD concentration (1.56 µg/mL) in the T + C1 mixture and the other with a higher CBD concentration (3.12 µg/mL) in the T + C2 mixture. The CI values for tiamulin and cannabidiol are presented in Table 1. The increase in the IC_50_ value for the mixture T + C1 compared to the effect of the drug alone indicated an antagonistic or agonistic nature of the interaction, especially in the MTT and TPC assays for SH-SY5Y cells, the LDH assay for HepG2 cells and the MTT assay for HEK-293 cells. Increasing the CBD concentration in the T + C2 mixture led to an increase in the IC_50_ value compared to the T + C1 mixture, indicating strong antagonistic interactions between the two compounds.

**Figure 4:**
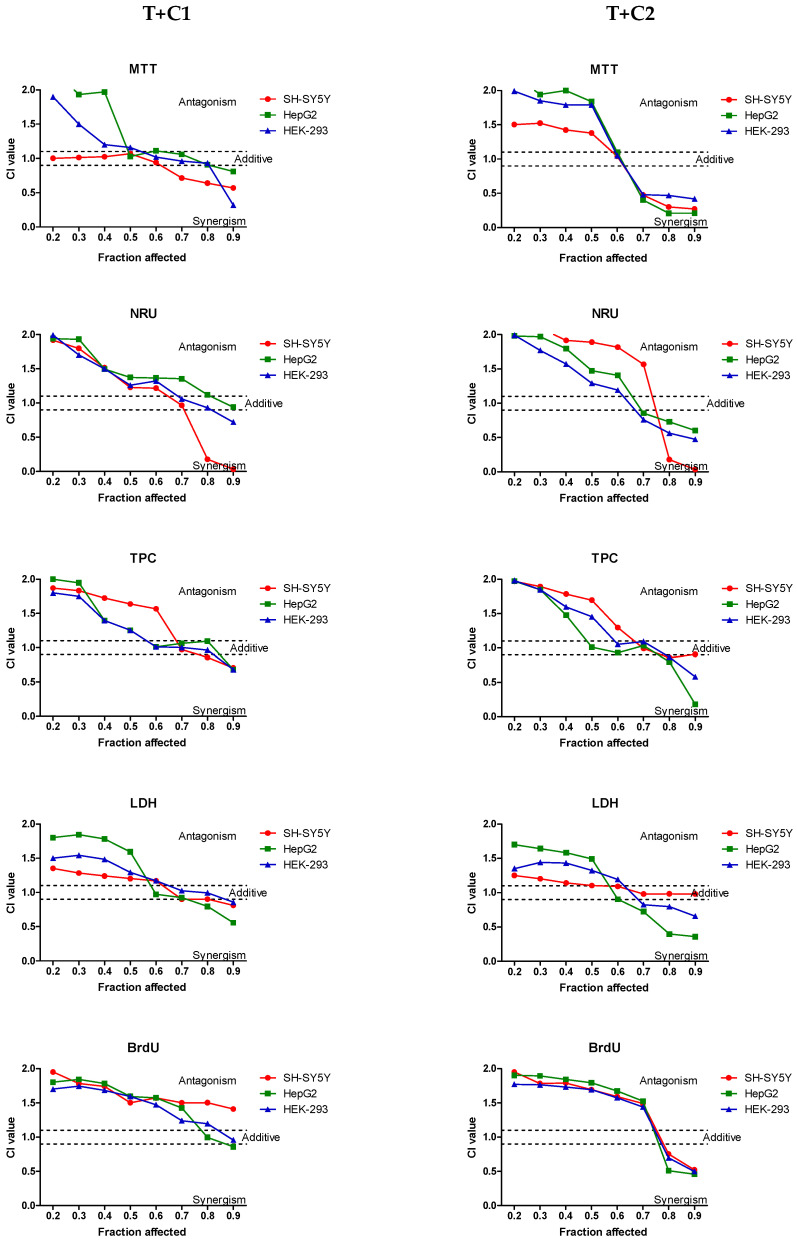
Combination index-fraction affected curves (CI-Plot) for the combination of tiamulin and cannabidiol at low concentrations (1.56 µg/mL) (T + C1) and higher concentrations of CBD (3.12 µg/mL) (T + C2) in SH-SY5Y, HepG2 and HEK-293 cells using five different assays. CI values were calculated from the data obtained from three independent experiments. The vertical bars indicate 95% confidence intervals for CI values based on sequential deletion analysis. Horizontal dashed lines indicate the additive level, which separates the synergism and antagonism sides.

Figure 4 shows the CI values calculated across a wide range of cytotoxicity levels (from 20% to 90%) for both combinations tested. The nature of the interactions depended on the concentration of tiamulin in both mixtures for all used cellular cultures. Strong antagonistic effects were observed at low drug concentrations, while synergistic effects were seen at high tiamulin concentrations in both mixtures with CBD. Differences between the two mixtures were primarily observed in the strength of the antagonistic and synergistic effects. For SH-SY5Y cells, the T + C1 mixture caused an additive effect in the MTT assay and very weak antagonistic effects in the LDH assay. In contrast, exposure of the cells to the T + C2 mixture at low tiamulin concentrations resulted in more pronounced antagonistic effects. Increasing the CBD concentration in the T + C2 mixture led to interactions with a strong antagonistic character at low tiamulin concentrations.

### 2.3. Reactive Oxygen Species (ROS) Detection

**Figure 5:**
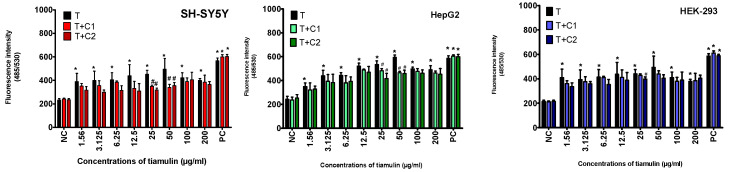
Evaluation of reactive oxygen species (ROS) by DCFH-DA assay after 72 h of treatment of SH-SY5H, HepG2 and HEK-293 cells with tiamulin and co-treatment with cannabidiol at concentrations of 1.56 µg/mL (T + C1) and 3.12 µg/mL (T + C2). Data are expressed as mean ± SD (standard deviation) of at least three experiments. (* *p* ≤ 0.05; # *p* < 0.01).

The effect of tiamulin and its two mixtures with cannabidiol (T + C1 and T + C2) on reactive oxygen species (ROS) production was studied. The results showed that ROS levels depended on both the cell line and the tiamulin concentration. The fluorescence intensity of DCF (2′,7′-dichlorofluorescein) in cells treated with tiamulin was higher than in cells treated with the drug mixtures containing cannabidiol. The effect of the veterinary drug alone induced stronger ROS production compared to its mixture with CBD. The increase in ROS production was proportional to the concentration of tiamulin. However, at the two highest concentrations of tiamulin (100 and 200 µg/mL), ROS levels decreased compared to the 50 µg/mL concentration. This decrease is likely due to the higher cell mortality at the higher drug concentrations (Figure 5).

At the lowest concentration of tiamulin, the fluorescence intensity of the probes increased by 66%, 44% and 91% in SH-SY5Y, HepG2 and HEK-293 cell cultures, respectively. When cells were exposed to T + C1 or T + C2, there was an approximate 10% or 20% decrease in ROS levels, respectively, compared to the lowest concentration of tiamulin alone (Figure 5). These results suggest that cannabidiol protect cells from oxidative stress by reducing ROS levels.

### 2.4. Apoptosis and Necrotic Profiles of Cells

**Figure 6:**
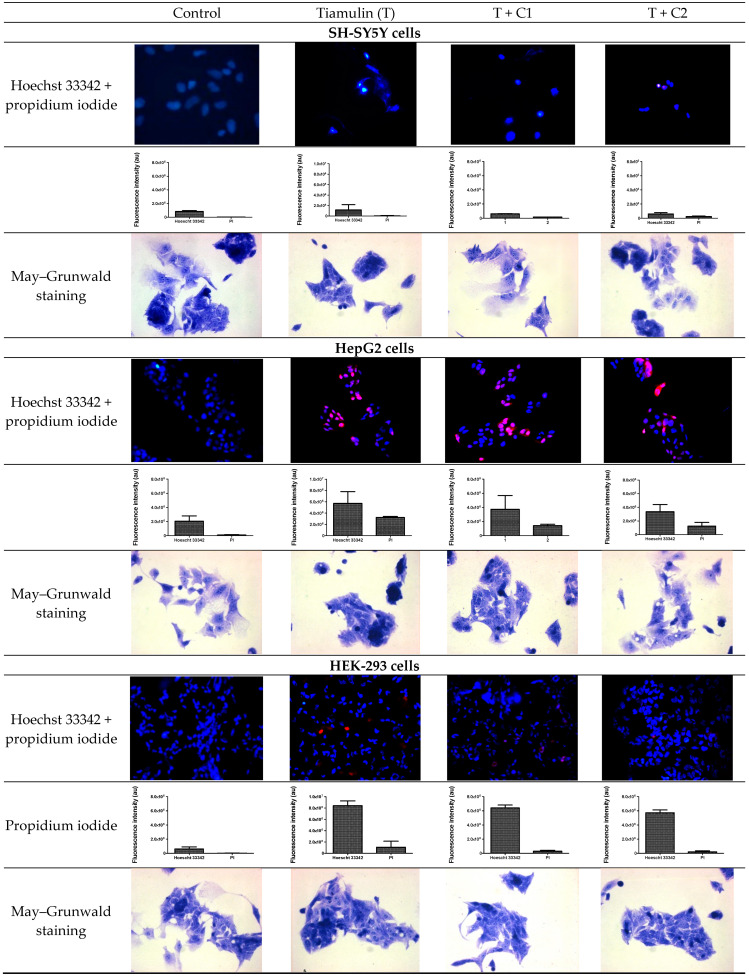
Representative fluorescence images of Hoechst 33342+ propidium iodide for each group. Column graphs show median fluorescence intensity. Morphological evaluation of SH-SY5Y, HepG2 and HEK-293 cells (control vs. treatment: T (tiamulin); T + C1 (tiamulin with cannabidiol at 1.56 µg/mL); T + C2 (tiamulin with cannabidiol at 3.125 µg/mL), 72 h of incubation) using May–Grunwald staining (200×).

After assessing the cytotoxicity of tiamulin and its mixtures with CBD, cell death was examined using the Hoechst 33342/propidium iodide (PI) double staining test (Figure 6). The intensity of the blue color and the presence of fragmented, blue-stained cellular components indicated apoptotic cell death after Hoechst 33342 staining. Necrotic cell death was identified by red fluorescence after PI staining. A slight increase in apoptotic and necrotic cells was observed in SH-SY5Y neuronal cells after exposure to tiamulin. Mixtures of tiamulin and CBD caused a decrease in both apoptotic and necrotic cell death. Tiamulin induced significant apoptotic and necrotic profiles in HepG2 and HEK-293 cells. Depending on the concentration of CBD in the mixture, a reduction in apoptotic and necrotic cells was observed in both HepG2 and HEK-293 cultures.

### 2.5. Morphological Assessment of Cells

Analysis of cell cultures after exposure to tiamulin and its mixture with CBD revealed changes in cell morphology in SH-SY5Y, HepG2 and HEK-293 cells (Figure 6). It was observed that tiamulin affected cell growth by causing detachment from the adhesion surface. Additionally, rounding of the cells and their separation from each other were noted. The cells shrank, leaving only a thin cytoplasmic connection between neighboring cells.

## 3. Discussion

Animal breeding relies heavily on the use of veterinary drugs. However, common mistakes made by breeders can lead to the presence of these drugs in edible animal tissues, creating a source of exposure for consumers [5,13]. This study shows that long-term (72 h) exposure of human cells to tiamulin causes neurotoxic effects. At the lowest concentration of 1.56 µg/mL, the drug inhibited lysosomal activity, followed by inhibition of mitochondrial activity and DNA synthesis. In HepG2 cells, toxic effects were observed at a concentration of 12.5 µg/mL, where mitochondrial activity was inhibited. This concentration of tiamulin in human HEK-293 cells led to the inhibition of DNA synthesis, thereby affecting cell proliferation. Other studies have shown cytotoxic effects of tiamulin after 10 h of exposure at concentrations of 100 µM (49.4 µg/mL) and 200 µM (98.8 µg/mL) in HepG2 and HEK-293 cells [31,32]. The cytotoxic effects of tiamulin have also been evaluated in RAW264.7 macrophages. No significant cytotoxicity was observed for tiamulin at concentrations less than 5 μg/mL. The drug at concentrations up to 20 μg/mL did not result in a <50% reduction in the macrophages’ viability [33]. Cytotoxicity studies of tiamulin on other human cells (MDA-MB-231) showed that cell viability decreased with increasing dose (6.25–100 µg/mL) and treatment period (12–48 h). The results indicated that the drug exerted its effect in a clearly cell-, dose- and time-dependent manner [34], indicating the mechanism of tiamulin toxicity may involve the inhibition of translation initiation in the mitochondria of HEK-293 cells, likely through the same mechanism as in prokaryotes. The drug binds to the peptidyl transferase center (PTC) of the ribosome, disrupting the positioning of the initiator tRNA and the A site of the ribosome, causing the inhibition of protein synthesis [35]. Based on the IC_50_ concentration values, it can be indicated that the mechanism of action of tiamulin in human HepG2 and HEK-293 cell lines was associated with the inhibition of mitochondrial activity leading to the inhibition of DNA synthesis and thus cell proliferation. However, a different mechanism was found in nerve cells, in which the drug inhibited lysosomal activity and cellular DNA synthesis. It should be noted that tiamulin, at the lowest concentration tested, increased the production of intracellular reactive oxygen species (ROS) in all cell cultures used, indicating a damaging effect at the subcellular level. The results of our previous study on primary porcine hepatocytes showed that tiamulin is a potent inducer of ROS, leading to cytotoxic effects. At a concentration of 250 µM (123.5 µg/mL), tiamulin increased ROS production in the hepatocyte culture after 24 h of exposure [36]. Additionally, it should be noted that research has shown the cytotoxicity of tiamulin leads to changes in cell morphology, which was further confirmed by an increase in apoptotic and necrotic cell death in the tested cultures.

Based on our research results, it can be concluded that tiamulin can lead to health disorders in humans. Therefore, the next stage of this research will involve minimizing the toxic effects of this veterinary drug in consumers. An important aspect is that tiamulin can cause toxic interactions with other drugs/substances used at the same time. It has been proven that concomitant use of tiamulin with certain ionophore antibiotics (e.g., monensin, salinomycin) can cause fatal poisoning in animals. Tiamulin affects liver cytochrome P450 activity by inhibiting the N-demethylation of ethylmorphine and the hydroxylation of testosterone. Selective inhibition of cytochrome P450 enzymes from the CYP4503A subfamily has been demonstrated. Therefore, potential toxic interactions between tiamulin and drugs or endogenous compounds metabolized oxidatively by CYP450 enzymes should be considered [37,38].

The widespread public interest in and increased sales of hemp-based products and their growing therapeutic applications, including confirmed neuro-, hepato- and nephroprotective effects, have pointed to their potential use in these studies. In this study two concentrations of CBD (1.56 and 3.125 µg/mL) were applied, which in previous studies did not show a decrease in cell viability. CBD exhibited cytoprotective effects on cells, alleviating the toxic impact of tiamulin. Data from the literature confirm the protective action of cannabidiol against the cytotoxicity of drugs or toxins. CBD has demonstrated therapeutic potential in toxic liver damage induced by CCl_4_, cadmium or alcohol [20,39,40]. The innovative results of our research indicate the protective effect of cannabidiol on tiamulin-induced cytotoxicity by reducing oxidative stress and exhibiting anti-apoptotic effects. Additionally, the evaluation of the interaction between CBD and tiamulin indicates the protective action of CBD. The antagonistic nature of the interaction between tiamulin and CBD demonstrated in this study was confirmed by the increase in the IC_50_ value of the mixture of tiamulin and CBD. This increase in IC_50_ depended on the concentration of CBD, with the strength of the antagonistic (protective) effect increasing accordingly. The antagonistic action (reducing the cytotoxicity of the drug) can be explained by the effective scavenging of free radicals by CBD, which leads to a reduction in ROS production in the cell cultures studied. Data from the literature confirm the effective protection of liver function, reduced oxidative stress and inhibition of pro-inflammatory factor production [20,39,40]. However, these interactions were further analyzed based on the drug concentration in the culture, showing that the protective (antagonistic) effect of cannabidiol was most pronounced at low concentrations of tiamulin. As the tiamulin concentration increased, the interaction shifted towards a synergistic effect, increasing the drug’s cytotoxicity. The likely increase in ROS production at high concentrations of the veterinary drug and the resulting increase in cell mortality may contribute to exceeding the antioxidant capacity of CBD.

Cannabidiol has been shown to exert hepatoprotective effects through the regulation of Nrf2 and antioxidant enzymes, leading to a reduction in apoptosis [41]. In our study, we demonstrated strong antioxidant and anti-apoptotic effects of CBD, which were dependent on the CBD concentration in the mixture with the veterinary drug tiamulin. Furthermore, CBD exhibited anti-apoptotic effects and prevented changes in cell morphology. Based on data from the literature, the protective effect of CBD on tiamulin-induced cytotoxicity can also be explained by its action on various cytochrome P-450 enzymes. CBD has been shown to be the strongest inducer of CYP1A1 expression [42]. On the other hand, tiamulin strongly blocks CYP3A4, leading to toxic interactions with drugs also metabolized by this cytochrome [8]. This mechanism has not been studied; however, this topic requires further metabolic research.

The hepatoprotective and neuroprotective effects of CBD obtained in this study are also supported by data from the literature indicating other protective mechanisms beyond antioxidant activity. Studies have shown that CBD may have a protective effect on the liver and brain from the toxic effects of cocaine or alcohol, minimizing inflammatory damage [40,43]. CBD reduces alcohol-induced liver damage by decreasing lipid accumulation and oxidative stress, stimulating autophagy and modulating the inflammatory response. CBD also reduces alcohol-induced brain damage by preventing neuron loss through its antioxidant and immunomodulatory properties. CBD also exerts a modulatory effect on antioxidant enzymes, restoring normal levels of SOD and CTL in the nervous system after exposure to aluminum [44]. Considering the numerous beneficial effects resulting from the pleiotropic mechanisms of CBD, which contribute to its cytoprotective action, several should be distinguished. One of these mechanisms is CBD’s reduction of oxidative stress and the reduction of reactive oxygen and nitrogen species (ROS/RNS). Among other effects, superoxide anion levels are reduced due to increased superoxide dismutase (SOD) and glutathione peroxidase induced by CBD. Other antioxidant enzymes, such as catalase (CAT), which catalyzes the conversion of H_2_O_2_ to water, can protect cells from oxidative stress [45]. Another protective mechanism of CBD is the reduction of pro-inflammatory cytokine expression, such as IL-6 and IFN-α, as well as the production of matrix metalloproteinases (MMPs) [46]. CBD also plays an important role in maintaining ion balance under stress conditions; for example, it can modulate the Na^+^/Ca^2+^ exchanger and promote calcium storage. CBD administration also inhibits apoptosis and supports cell survival. During cell survival, increased HO-1 expression may play a key role [47]. Numerous reports show that, unlike other cannabinoids, CBD has weak effects on cannabinoid receptors 5 [48]. However, CBD activates transient receptor potential (TRP) channels, such as TRPA1, TRPV1 and TRPV4, even at nanoparticle concentrations [49]. The next research project will take into account these mechanisms in evaluating the protective effect of CBD on tiamulin-induced cytotoxicity.

The nephroprotective effect of CBD in relation to tiamulin toxicity indicates its antioxidant and anti-apoptotic actions. The literature also suggests additional mechanisms of nephroprotective action. It has been shown that CBD treatment significantly weakens the oxidative/nitrosative stress induced by cisplatin, inflammation and cell death in the kidneys, as well as improves kidney function [50]. Studies have shown that CBD suppresses the mRNA of pro-inflammatory cytokines in cisplatin-induced nephropathy and significantly reduces apoptosis by inhibiting caspase-3 activity [51]. The impact of CBD on drug-induced nephropathies is currently the subject of numerous studies aimed at determining the mechanism of CBD’s action [52].

Data from the literature indicate the other positive effects of CBD when combined with other concurrently used drugs. CBD reduced neurotoxicity induced by PTX through 5-HT1A receptors without affecting the functioning of the nervous system or the effectiveness of chemotherapy [53]. CBD improved cardiomyopathy induced by DOX through the regulation of heart mitochondrial function in mouse models [54]. An antagonistic interaction in terms of anti-proliferative effects against melanoma cells between CBD and anticancer drugs (mitoxantrone or cisplatin) has been shown [55] without indicating the protective mechanism of CBD. Other studies on the effectiveness of CBD in veterinary chemotherapy showed that combinations of CBD with mitoxantrone and vinblastine showed a synergistic interaction through a significant reduction in cell viability and increased apoptosis in canine urothelial cancer. In contrast, the combination of cannabidiol and carboplatin showed an antagonistic effect, which did not reduce cell viability or increase apoptosis. Moreover, the combination of the non-steroidal anti-inflammatory drug piroxicam with cannabidiol had no significant impact on the viability of urothelial cancer in dogs [56].

It should be noted that the use of CBD in veterinary medicine is increasing, as well as in public health protection. However, further studies are needed in this area to precisely determine the mechanism of action of veterinary drugs on human cells, taking into account other cell models and to consider the interaction of CBD in light of its protective mechanisms. It should be taken into account that interactions between CBD and residues of veterinary drugs in food have not been studied. Therefore, further research is necessary to assess the impact of CBD on veterinary drugs present in food over longer exposure periods for consumers.

## 4. Materials and Methods

### 4.1. Materials

Analytical standards of tiamulin fumarate (CAS: 55297-96-6; molecular weight: 609.8 g/mol nr cat. 46959 Sigma, Poznan, Poland) and cannabidiol (CAS: 13956-29-1; molecular weight: 314.5 g/mol nr cat. 85705 PhytoLab, Poznan, Poland). Triton X-100, hydrogen peroxide, trypan blue, dimethyl sulfoxide (DMSO), fetal bovine serum (FBS), bovine calf serum (BCS), neutral red dye (NR), Coomassie brilliant blue R-250 dye, 3-(4,5-dimethylthiazol-2-yl)-2,5-diphenyltetrazolium bromide (MTT), trypsin-ethylenedinitrilotetraacetic acid (EDTA) and antibiotic solution (10,000 U/mL of penicillin, 10 mg/mL of streptomycin) were purchased from Sigma–Aldrich Co. (Poznan, Poland). All other chemicals were purchased from commercial suppliers and were of the highest available purity.

### 4.2. Cell Line Cultures

The human neuroblastoma (SH-SY5H) [27,28], hepatoma (HepG2) [29,30] and kidney cells (HEK 293) [51,52] cells were purchased from the American Type Culture Collection: ATCCCRL-2266,ATCC HB-8065 and ATCC CRL-1573, respectively. These cells were cultured in Minimum Essential Medium Eagle (MEME) (ATCC). The media were supplemented with 10% FBS, 1% L-glutamine (and 1% antibiotic solution (P4333 Sigma). The cells were maintained in 75 cm^2^ cell culture flasks (NUNC) in a humidified incubator at 37 °C in an atmosphere of 5% CO_2_. The medium was refreshed every two or three days, and the cells were subcultured by 0.25% trypsin–0.02% EDTA after reaching 70–80% confluence. Single cell suspensions were prepared and adjusted to a density of 2 × 10^5^ cell/mL^−1^ (SH-SY5H), 1 × 10^5^ cell/mL^−1^ (HepG2) and 1 × 10^5^ cell/mL^−1^ (HEK-293). The cell suspension was transferred to 96-well plates (100 µL/well) or Lab-Tek chamber slides (8 wells) (Nunc) for staining and incubated for 24 h before the exposure to the studied compounds. Stock solutions of tiamulin fumarate and cannabidiol were prepared in DMSO and diluted with culture medium to obtain a concentration range from 1.56 to 200 µg/mL and 1.56 or 3.156 µg/mL, respectively. The selection of tiamulin concentrations was based on data from the literature [10] and pilot studies. The final concentration of DMSO was 0.1% in the medium. The same final concentrations of the solvent and the 1% Trition-X 100 solution were used as the negative and positive controls, respectively. A total of 500 µM hydrogen peroxide (H_2_O_2_) in was used as the positive control in the DCFH assay. The medium used for test solutions and in the control preparation did not contain serum and antibiotics. As a negative control, cultured cells were grown in the absence of studied compounds. Each concentration was tested in six replicates in three independent experiments. Cytotoxicity was assessed after 72 h of exposure of the cells to the compounds. The medium was not changed during the incubation time.

### 4.3. MTT Assay

The metabolic activity of living cells was assessed by the measurement of the activity of dehydrogenases [57]. After the incubation of the cells with the studied compounds, 10 μL of the MTT solution (5 mg/mL in PBS) was added to each well of 96-well plates and incubated. After 3 h, the MTT solution was removed, and the intracellular formazan crystals were dissolved in 100 µL DMSO. The plate was shaken for 15 min at room temperature and transferred to a Synergy 2 Multi-Mode Microplate Reader (BioTek^®^ Instruments Inc., Winooski, VT, USA) to measure the absorbance at 570 nm, using a blank as a reference. Cytotoxicity was expressed as a percentage of the negative control (0.1% DMSO in medium) [58].

### 4.4. NRU Assay

This method is based on staining living cells with neutral red, which readily diffuses through the plasma membrane and accumulates in lysosomes [59]. After the incubation, the medium containing the tested substance was removed and the cells were washed with PBS. Then, 100 µL/well of NR solution (50 µg/mL) was added for 3 h. After this time, the cells were washed with PBS. The dye from viable cells was released by extraction with a mixture of acetic acid, ethanol and water (1:50:49, *v*:*v*:*v*). After 10 min of shaking, the absorbance of the dissolved NR was measured using a Synergy 2 Multi-Mode Microplate Reader (BioTek^®^ Instruments Inc., Winooski, VT, USA) at 540 nm using a blank as a reference. Cytotoxicity was expressed as a percentage of the negative control (0.1% DMSO in medium) [58].

### 4.5. TPC Assay

This assay is based upon staining the total cellular protein (proliferation) [60]. After the incubation, the medium containing the drug was removed, and 100 µL Coomassie brilliant blue R-250 dye was added to each well. The plate was shaken for 10 min. Then, the stain was removed and the cells were rinsed twice with 100 µL of washing solution (glacial acetic acid/ethanol/water, 5:10:85, *v*:*v*:*v*). After that, 100 µL of the desorbing solution (1 M potassium acetate) was added, and plates were shaken again for 10 min. The absorbance was measured at 595 nm in a Synergy 2 Multi-Mode Microplate Reader (BioTek^®^ Instruments Inc., Winooski, VT, USA) using a blank as a reference. Cytotoxicity was expressed as a percentage of the negative control (0.1% DMSO in medium) [58].

### 4.6. LDH Leakage Assay

The integrity of the plasma membrane was assessed through the test of lactate dehydrogenase (LDH) release [61], which was monitored using the commercially available Cytotoxicity Detection Kit (LDH) (Roche Diagnostics, Warsaw, Poland). The medium (100 µL/well) without cells was transferred into the corresponding wells of an optically clear 96-well flat bottom microplate, and 100 µL reaction mixture was added to each well. Then, the plates were incubated for 30 min at room temperature in darkness. After that time, 50 µL/well of 1 M HCl was added to stop the reaction. The absorbance was measured at 492 nm in a Synergy 2 Multi-Mode Microplate Reader (BiTek^®^ Instruments Inc., Winooski, VT, USA) using a blank as a reference [58].

### 4.7. BrdU Assay

Cell proliferation was evaluated by BrdU assay using a Cell Proliferation ELISA, BrdU (colorimetric) kit (Roche, Switzerland), according to manufacturer’s instructions. Briefly, BrdU was incorporated in the place of thymidine into the DNA of cells under division. This BrdU was detected by an anti-BrdU antibody conjugated with peroxidase and the reaction product of the enzyme with the given substrate was quantified by measuring its absorbance. The absorbance (A) was measured at 370 nm and 492 nm in a Synergy 2 Multi-Mode Microplate Reader (BioTek^®^ Instruments Inc., Winooski, VT, USA), using a blank as a reference.

### 4.8. DCFH Assay

Intracellular levels of ROS were evaluated using the redox-sensitive fluorescent dye DCFH-DA [62]. In brief, the cells were incubated with 5 μM DCFH-DA for 1 h at 37 °C in the dark. Thereafter, the tested compounds at study concentrations were added. The cells treated with 500 µM hydrogen peroxide (H_2_O_2_) were taken as a positive control. After 72 h of incubation, the fluorescence of 2′,7′-dichlorofluorescein (DCF) was measured. Fluorescence was determined at 485/530 nm using a fluorescence microplate reader (BioTek^®^ Instruments Inc., Winooski, VT, USA).

### 4.9. Apoptosis and Necrosis Identification

The analysis of type and level of cellular death in the control and tested cultures was evaluated using fluorochrome staining, Hoechst 33342 (Sigma, St. Louis, MO, USA) and propidium iodide (Sigma, St. Louis, MO, USA) mixed for apoptosis and necrosis evaluation [63]. Using an Axiaert 200 M fluorescence micrscope (Zeiss, Obercochem, Germany), dead cells were analyzed morphologically. Cells with pink fluorescent nuclei were considered necrotic, whereas those with blue fluorescent nuclei (fragmented and/or with condensed chromatin) were considered apoptotic.

### 4.10. Cellular Morphology Analysis, May–Grünwald–Giemsa (MGG) Staining

This method was used to illustrate the changes that occurred in cell morphology under the influence of the tested compounds. The staining was performed on 24-well plates. The cells at a density of 1 × 10^5^ cells/mL were used. After 24 h of incubation with the examined drugs or without them (control), the culture media was removed and the cells were stained with 1 mL of May–Grünwald dye for 3 min at room temperature. Then, 1 mL of deionized water was added to each plate. After 3 min of incubation at room temperature, the liquid was removed. All the plates were rinsed with deionized water, and then the cells were stained with Giemsa dye (dilution 1:20) for 30 min at room temperature. After this time, the dye was removed and the wells were rinsed with 1 mL of deionized water and then allowed to dry. The images were taken using a light microscope (Zeiss, Obercochem, Germany).

### 4.11. Isobolograms Method for Interactions Assessment

In order to determine the types of interaction that occur when cells are exposed to combinations of ENRO and DON, a method established by Chou and Talalay [64] was chosen in this study. The dose–effect relationships of the individual and combined study substances were biometrically modeled using the Median-Effect Equation of the Mass Action Law as described.

fa/fu = (D/Dm)m(1)

D—dose of the drug or CBD; fa—fraction affected by D; fu—fraction unaffected (i.e., fu = 1 − fa); Dm—median-effect dose (e.g., IC_50_); m—coefficient signifying the shape of the dose–effect relationship (m = 1, m > and m < 1 indicated hyperbolic, sigmoidal and flat sigmoidal dose–effect curves, respectively).

In this isobolographic analysis, combination index (CI) is a quantitative parameter used to evaluate the type of interactions and the corresponding influential level among multiple compounds. For all combinations, CI values were generated over a range of fractions of cell viability affected (Fa) from 0.05–0.97 (5–95% toxicity). CI = 0.9–1.1, CI < 0.9 and CI > 1.1 indicate additive, synergistic and antagonistic effects, respectively.

### 4.12. Statistical Analysis

This study was performed in three independent experiments. The obtained results are presented as mean values ± SD (standard deviation). The assessment of the cytotoxicity data used a one-way analysis of variance (ANOVA) followed by Dunnett’s post hoc test, which was applied to determine significance relative to the negative control. The cytotoxicity concentration (IC_50_) necessary for a 50% inhibition of cell viability by the drug was calculated using GraphPad Prism 5.0. Statistical comparisons among IC_50_ values were performed with an analysis of variance (ANOVA) followed by the Tukey test, and differences were considered to be statistically significant at *p* ≤ 0.05.

## 5. Conclusions

The results of the present study indicate that cannabidiol provided protective effects against tiamulin-induced cytotoxicity in human cells. The antioxidant and anti-apoptotic activities of cannabidiol can be considered the main factors responsible for its cytoprotective effects. Therefore, cannabidiol may be a potential candidate for preventing tiamulin-induced cellular injury and dysfunction. Further studies considering other cellular models, other mechanisms of CBD’s protective action and nonclinical studies in animals are necessary to confirm the obtained in vitro results.

## Figures and Tables

**Table 1:** The inhibited concentration (IC_50_, µg/mL) values determined in SH-SY5Y, HepG2 and HEK 293 cells by the MTT, NRU, TPC, LDH and BrdU assays after 72 h exposure to tiamulin (T) and its co-incubation with cannabidiol at concentrations of 1.56 µg/mL (T + C1) and 3.12 µg/mL (T + C2); data are presented as mean ± SEM (n = 3).

Cell Lines	Assay	T	T + C1	CI	T + C2	CI
SH-SY5Y	MTT	30.2 ± 1.8 ^a^	30.8 ± 1.8 ^a^	0.95	58.5 ± 1.8 ^b^	3.45
	NRU	2.1 ± 0.4 ^a^	7.9 ± 1.3 ^b^	1.66	16.7 ± 1.4 ^c^	2.97
	TPC	169 ± 3.3 ^a^	162 ± 4.9 ^a^	0.91	>200	
	LDH	>200	>200		>200	
	BrdU	9.9 ± 1.1 ^a^	34.8 ± 2.4 ^b^	2.07	50.0 ± 1.2 ^c^	2.84
HepG2	MTT	27.4 ± 1.3 ^a^	28.7 ± 1.1 ^a^	1.19	79.1 ± 1.3 ^b^	3.17
	NRU	34.7 ± 1.0 ^a^	36.1 ± 1.0 ^a^	1.50	98.9 ± 0.8 ^b^	3.59
	TPC	26.7 ± 1.4 ^a^	63.7 ± 2.5 ^b^	3.54	74.0 ± 1.3 ^b^	2.31
	LDH	39.5 ± 1.7 ^a^	37.6 ± 0.7 ^a^	0.97	39.7 ± 0.3 ^a^	0.99
	BrdU	13.9 ± 0.8 ^a^	37.1 ± 0.8 ^b^	2.73	39.9 ± 0.7 ^b^	1.07
HEK 293	MTT	27.5 ± 0.8 ^a^	27.9 ± 0.8 ^a^	0.99	28.7 ± 1.8 ^a^	1.09
	NRU	34.5 ± 1.1 ^a^	43.8 ± 2.5 ^b^	2.63	92.3 ± 1.1 ^c^	3.16
	TPC	8.5 ± 0.6 ^a^	18.7 ± 1.6 ^b^	3.13	16.3 ± 1.3 ^b^	2.81
	LDH	76.9 ± 2.3 ^a^	171 ± 3.6 ^b^	3.97	>200	
	BrdU	14.6 ± 1.3 ^a^	41.9 ± 0.7 ^b^	3.53	42.2 ± 0.9 ^b^	3.13

The different small letters (a–c) within lines indicate significant differences between the drug (*p* ≤ 0.05). Combination index (CI): a quantitative measure of the degree of drug interaction in terms of additive effect (CI = 1), synergism (CI < 1) or antagonism (CI > 1) for a given endpoint of the effect measurement.

## Data Availability

Data are contained within the article.
